# Less is more: the role of preoperative ultrasound as a risk stratifying tool in identifying low volume axillary disease in breast cancer

**DOI:** 10.1093/bjr/tqag145

**Published:** 2026-06-09

**Authors:** Yasaman Salari, Bhavani Rengabashyam, Vahid Shafiei

**Affiliations:** Department of Breast Radiology, York and Scarborough Teaching Hospital NHS Foundation Trust, York YO31 8HE, United Kingdom; Department of Breast Radiology, York and Scarborough Teaching Hospital NHS Foundation Trust, York YO31 8HE, United Kingdom; Accident and Emergency Department, University Sussex Hospital NHS Foundation Trust, Worthing BN11 2DH, United Kingdom

**Keywords:** axillary ultrasonography, lymph node excision, sentinel lymph node biopsy, lymph node dissection, breast neoplasm

## Abstract

**Objectives:**

Axillary lymph node dissection (ALND) in early breast cancer causes lymphoedema in up to 20% of women and may be unnecessary in those with low axillary disease burden. This study evaluates whether preoperative axillary ultrasound (AUS) can stratify nodal burden to support targeted axillary dissection (TAD).

**Methods:**

A retrospective single-centre observational study was conducted using records from January 2023 to December 2024. Among 193 patients with abnormal AUS, those with negative axillary biopsy, neoadjuvant therapy, no surgery, or distant metastases were excluded. Data on AUS findings, tumor size, and postoperative nodal histology were collected. Differences between pN1 (<4 positive nodes on ALND) and pN2-3 (≥4 positive nodes on ALND) disease were analyzed using non-parametric and categorical tests.

**Results:**

Of 193 patients, 142 (73.6%) had positive axillary biopsies; 134 were included. Among these, 76 (56.7%) had 1-2 abnormal nodes on AUS. Of 77 evaluable cases, 33 (42.9%) had pN1 and 44 (57.1%) pN2-3 disease. The mean number of abnormal nodes was higher in pN2-3 (3.05 ± 1.85) than pN1 (1.52 ± 0.89; *P = .*0001). Tumor size was greater in pN2-3 (57.9 ± 33.7 mm) than pN1 (36.5 ± 24.3 mm; *P = .*0012). AUS performance: sensitivity 87.1%, specificity 55%, PPV 60%, NPV 84.6%, accuracy 69%.

**Conclusions:**

AUS effectively identifies patients with low-volume axillary disease preoperatively and can guide TAD, reducing unnecessary ALND.

**Advances in knowledge:**

Preoperative AUS with biopsy predicts low-volume nodal disease with high sensitivity and NPV, enabling accurate triage for TAD and avoiding overtreatment.

## Introduction

### Historical evolution of axillary management in breast cancer

Historically, axillary lymph node dissection (ALND) was the mainstay for axillary staging and control in early breast cancer.^1^ However, high rates of morbidity, including lymphoedema, chronic pain, sensory loss, and reduced arm mobility, led to a progressive shift toward less invasive approaches.^2^

The American College of Surgeons Oncology Group Z0011 (ACOSOG Z0011)^3^ and After Mapping of the Axilla Radiotherapy or Surgery (AMAROS)^4^ trials revolutionized axillary management by showing that ALND can be safely avoided without impacting long-term survival or locoregional recurrence significantly in women with T1-T2 invasive breast cancer and 1-2 positive sentinel lymph nodes. Subsequent studies have confirmed that axillary radiotherapy (ART) provides oncologic control equivalent to ALND with significantly reduced lymphedema risk and better arm function in this group.^3^^,^  ^5–7^

### Targeted axillary dissection: potentially safe and less morbid alternative

Targeted axillary dissection (TAD) is a surgical technique that may offer an effective alternative to ALND. This involves removing the biopsy-proven metastatic lymph node, which is usually localized using image guidance, along with the blue and or hot node following the sentinel lymph node procedure (SNB). This targeted approach is feasible and is associated with significantly fewer surgical complications than ALND.^8–10^ It also results in more accurate identification and removal of involved nodes than SLNB.^8–10^ In a cohort study of 199 node-positive breast cancer patients treated with systemic neoadjuvant therapy, clinical outcomes were assessed following TAD alone or TAD combined with ALND.^5^ After a median follow-up of 43 (38-48) months, no significant difference in recurrence or mortality was observed between patients who underwent TAD alone (*n = *119) and those who received additional ALND (*n = *80).^5^ TAD alone was associated with excellent outcomes, especially in patients who showed a good response to neoadjuvant systemic therapy (NST) and had at least 3 lymph nodes removed.^5^ These findings suggest that TAD can be a safe and effective alternative to ALND in selected patients.^5^ Furthermore, a recent study that compared TAD with ALND reported a lower lymphoedema rate and severity after TAD.^7^ This reduction in lymphoedema is clinically meaningful, as it translates into improved long-term arm function, reduced pain and swelling, and better overall quality of life for patients.

### Cost-effectiveness: lower burden on health systems

ALND incurs higher direct and indirect costs, including longer operative time, hospital stay, post-operative care, and long-term treatment for complications like lymphedema.^11^ Avoiding unnecessary ALND reduces workforce burden on rehabilitation services. This is especially relevant in resource-constrained settings, where optimizing treatment value is crucial.

### Rationale

While there is substantial evidence to support de-escalation of axillary surgery with clinically impalpable nodes, normal axillary imaging and 1 or 2 macro metastases on sentinel node biopsy and those patients with node positive disease showing good response to neoadjuvant chemotherapy, there is a mismatch in the management of patients who have upfront surgery for preoperative diagnosis of nodal metastasis.^3^^,^^4^^,^  ^6^ A 2022 survey of 54 UK breast units showed that ANC remains standard of care in node-positive breast cancer.^12^ One of the reasons for this is that patients with proven nodal disease preoperatively are thought to have a higher burden of axillary disease.^13^ Ultrasound (US) of the axilla, along with needle biopsy of suspicious nodes, is widely used for axillary staging. But the predictive value of US-detected, biopsy-proven abnormal lymph nodes in predicting low-burden axillary disease (<4 metastatic nodes) is not fully defined.^14^^,^^15^

Evidence from deescalation trials is continuously changing the axillary management landscape in breast cancer. The currently open national trial called Targeted Axillary Dissection versus Axillary Node Clearance in patients with Positive axillary Lymph nodes in Early breast cancer (TADPOLE)^16^ is yet another example of this trend. Against this backdrop, our study aims to generate evidence supporting the use of US as a risk stratifying tool in the preoperative prediction of those patients who are likely to have low volume disease in the axilla. By demonstrating the ability of preoperative US to distinguish pN1 from higher-burden (pN2/3) disease, our study could potentially support breast units to participate in the TADPOLE trial. However, our study does not evaluate treatment safety of TAD but instead examines whether AUS can provide clinically useful preoperative information to support patient selection for evaluation of de-escalation strategies. The oncological safety of replacing ALND with TAD requires validation in prospective trials, such as TADPOLE.^16^

## Methods

### Study design

This is a retrospective cohort study conducted at The York Hospital. The study was registered with the Trust’s governance team. It was deemed exempt from ethical approval due to its nature as an audit of routinely collected data (Registration Number: CSCS/BRE/096).

### Study period and population

Newly diagnosed screen-detected and symptomatic breast cancer patients with abnormal AUS findings were identified from electronic patient records (Somerset Cancer Register) during the period from January 2023 to December 2024.

#### Method

All US examinations have been performed by 3 consultant radiologists, 4 consultant radiographers, and 2 advanced practitioners with more than 5 years’ experience each. Philips EPIQ 5 W YU8 US machine with eL18-4 linear array transducer has been used in all patients. The patient lies supine with ipsilateral arm abducted 90 degrees and externally rotated (hand behind head). A systematic survey of all 3 nodal levels in the axilla is performed. The axilla is scanned from the level of the nipple in the lower axilla to the humeral head. Level 2 nodes are evaluated with transducer angled behind the pectoralis muscle; level III nodes are assessed via infraclavicular approach. Internal mammary nodes are not assessed routinely. Abnormal findings on AUS are defined as lymph nodes with a cortical bulge greater than 3 mm or loss of the medullary fatty hilum. The most abnormal node is chosen for biopsy under US guidance using a 14G Trucut needle and typically 2 core samples are obtained. It is routine practice to perform axillary biopsies under image guidance only and 16G or 18G Temno needle is used when the node is difficult to access.

### Inclusion and exclusion criteria

Patients were eligible for inclusion if they had non-palpable, abnormal biopsy proven metastasis in the axillary nodes and underwent ALND as the reference standard for nodal staging. Pathological nodal burden (pN1 vs. pN2-3) was determined based on the number of lymph nodes containing macro metastases (>2 mm metastatic deposit) identified on final histological assessment of the ALND specimen.

Exclusion criteria comprised patients who had received neoadjuvant systemic therapy, those deemed medically unfit for surgery due to frailty or the presence of metastatic disease at presentation, and patients who elected to decline surgical management.

It is routine practice to remove level I and II axillary lymph nodes in our unit in patients undergoing ALND. The total number of nodes retrieved at surgery was recorded, and nodal yield is reported as median, interquartile range (IQR), and range. TAD was defined as removal of the biopsy-proven metastatic lymph node (typically clipped at the time of diagnosis and localized prior to surgery) in combination with sentinel lymph nodes (SLNs) identified using dual localization techniques with methylene blue and radiotracer. The aim was to remove at least 4 nodes including the clipped node.

### Data collection

Data were collected from electronic medical records. US reports were reviewed to document the number of abnormal axillary lymph nodes identified. Axillary core needle biopsy results, SLNB results, operation notes, and multidisciplinary team (MDT) letters were examined to determine whether patients underwent additional axillary surgery, targeted axillary dissection (TAD), SLNB alone, or axillary node clearance (ANC).

Histopathology reports were used to extract data on tumor type, size, presence of lymphovascular invasion (LVI), nature of lymph node metastasis (micro- or macrometastasis), and receptor status (ER, PR, HER2). The number of lymph nodes involved following surgery was also recorded. Additionally, the source of referral (screen-detected vs. symptomatic presentation) was noted.

This was a retrospective study. AUS findings, including nodal count and morphology, were recorded as part of routine clinical care. The relationship between AUS findings and pathological nodal burden was analyzed retrospectively and did not influence surgical decision-making within the study.

### Study endpoints

The primary endpoint was to evaluate the diagnostic performance of AUS alone in correctly identifying patients with pathologic pN1 disease (ie, 1-3 involved lymph nodes on ANC) in cases with a biopsy-proven abnormal node. The secondary endpoint was to assess whether tumor characteristics (such as lymphovascular invasion also known as LVI, size, receptor status, or histological subtype) were associated with nodal disease burden.

### Statistical analysis

Descriptive statistics were used to summarize patient demographics and tumor characteristics. Diagnostic accuracy metrics such as sensitivity, specificity, positive predictive value (PPV), and negative predictive value (NPV) were calculated for AUS in identifying pN1 status. Associations between tumor characteristics and nodal disease burden were evaluated using appropriate inferential tests: Chi-square or Fisher’s exact test for categorical variables, and *t*-test or Mann-Whitney *U* test for continuous variables, as applicable. Statistical significance was defined as *P < .*05. All analyses were performed using Stata MP version 19.5. All statistical analyses were calculated and reported with 95% confidence intervals (CIs).

The predictors included in the regression model (US-detected nodal count and tumor size) were selected a priori based on clinical plausibility and prior literature demonstrating their association with axillary nodal burden.^17^^,^^18^ Tumor size was categorized using a clinically relevant threshold of ≤50 mm versus >50 mm, corresponding to T1-T2 and T3 disease according to established staging systems.^18^ Thresholds for categorization (1-2 vs. >2 abnormal nodes and ≤50 mm vs. >50 mm tumor size) were chosen to reflect clinically relevant decision-making groups rather than data-driven optimization.

## Results

A total of 193 patients with abnormal AUS findings were identified between January 2023 and December 2024. Of the 193 patients, 142 (73.6%) had a positive core needle biopsy. This included 78 patients in the 2023 cohort and 64 patients in the 2024 cohort. In the 2023 cohort, 28 patients were excluded because they had received neoadjuvant treatment (NAT), leaving 50 patients. Of these, 10 were further excluded due to metastatic disease at presentation, and one due to significant frailty did not have surgery, resulting in 39 patients included in the final analysis. In the 2024 cohort, 25 patients were excluded because they received NAT. One additional patient was excluded due to the presence of metastatic disease at presentation, yielding 38 patients included in the final analysis. In total, 77 patients met the inclusion criteria and were included in the final analysis (as shown in Figure 1).

**Figure 1:**
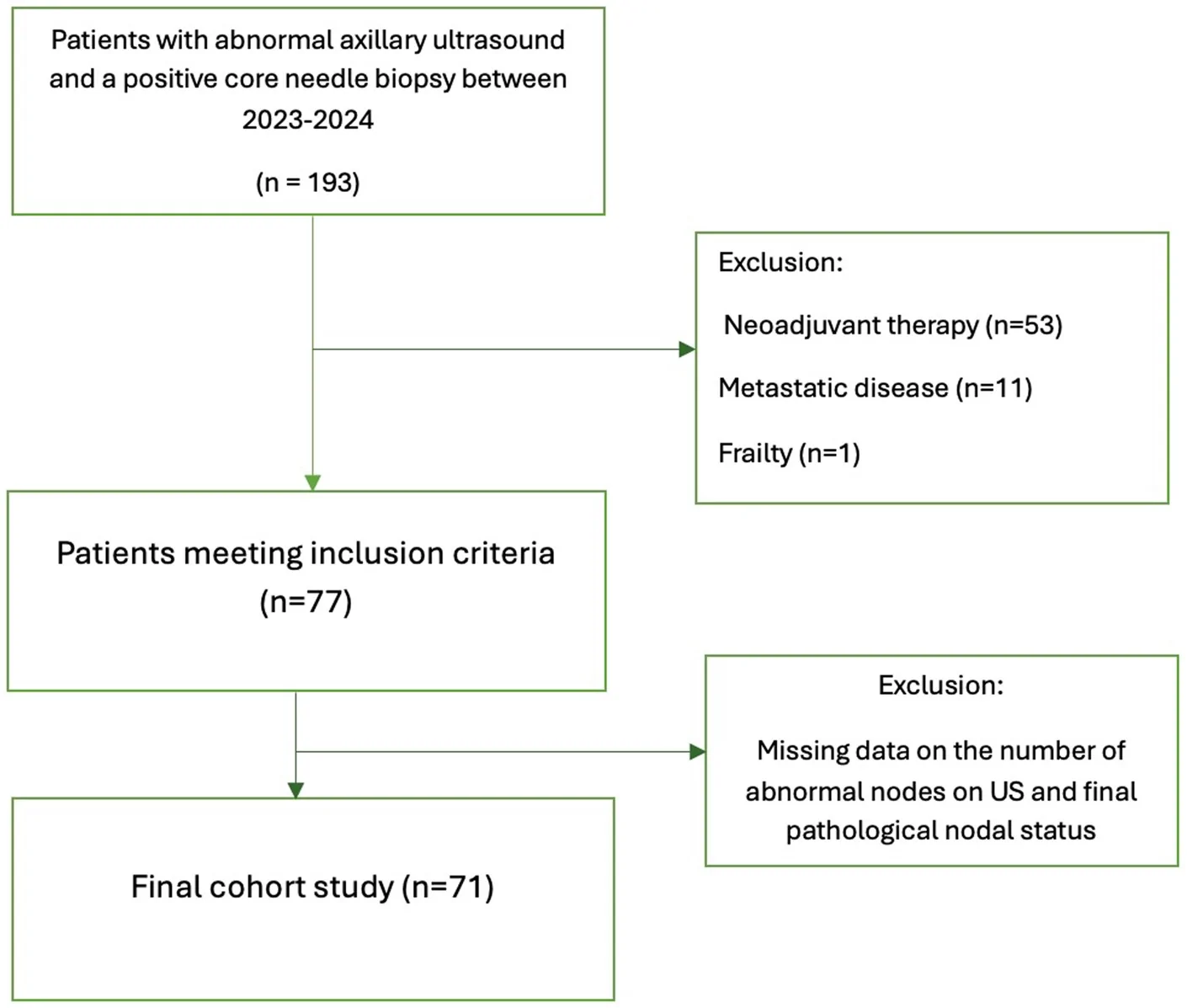
CONSORT-style flow diagram of patient selection. Patients with abnormal axillary ultrasound (US) findings and a positive core needle biopsy were identified from 2 consecutive cohorts (2023 and 2024). Patients were excluded if they received neoadjuvant treatment, had metastatic disease, or were deemed unsuitable due to frailty. After applying exclusion criteria 77 patients were included from which 6 had missing data on the number of abnormal nodes on US and final pathological nodal status which were excluded and the final cohort included 71 patients.

### Patients’ characteristics

All 77 patients were female and of Caucasian ethnicity. The mean age was 64.1 years (range, 31-83 years). Seventeen cases (22.1%) were screen-detected, while the remaining 60 (77.9%) presented symptomatically. The mean (SD) final pathological tumor size was 49.2 (31.8) mm. On final nodal staging, 33 patients (42.9%) were classified as pN1 and 44 (57.1%) as pN2/3. The median number of lymph nodes removed at axillary surgery was 18 (IQR 14-24, range 2-42).

The number of suspicious nodes was documented in 71 patients (92.2%), and the type of nodal abnormality was described on US in 45 patients (58.4%). Cases with missing data on US-detected nodal count (*n = *6) were excluded from analyses requiring this variable, and a complete-case analysis approach was used. Given the small proportion of missing data, no imputation was performed.

Histologically, pure invasive ductal carcinoma of no special type (NST) was the predominant subtype, accounting for 85.7% of cases. Pure invasive lobular carcinoma represented 7.8%, while rare subtypes, including micropapillary, metaplastic, mucinous, and mixed NST with lobular histology, each comprised 1.3% of cases. One case (1.3%) demonstrated ductal carcinoma in situ (DCIS) without invasive disease.

Most tumors were unifocal (80.3%), and lymphovascular invasion (LVI) was present in 58.4% of cases. Regarding receptor status, the majority were hormone receptor-positive and HER2-negative (76.6%), followed by triple-negative (9.1%), triple-positive (3.9%), and other profiles (10.4%). These characteristics are summarized in Table 1.

**Table 1:** Baseline characteristics of the study cohort (*N = *77).

Characteristic	Value
**Gender**	Female: 77 (100%)
**Race**	Caucasian: 77 (100%)
**Age, years**	Mean: 64.1. Range: 31-83.
**Type of referral**	Screen-detected = 17 (22.1%), symptomatic = 60 (77.9%)
**Final pathology tumor size, mm**	Mean (SD): 49.2 (31.8)
**PN stage**	pN1: 33 (42.9%), pN2/3: 44 (57.1%)
**Type of abnormality of lymph nodes reported on the AUS**	Reported: 45 (58.4%), not reported: 32 (41.6%)
**Reported number of suspicious lymph nodes on US**	Reported in 71 patients (92.2%), missing in 6 patients (7.8%)
**Histological subtype**	Pure invasive ductal NST: 66 cases (85.7%), pure lobular: 6 cases (7.8%), pure micropapillary: 1 case (1.3%), pure metaplastic: 1 case (1.3%), pure mucinous: 1 case (1.3%), mixed NST + lobular: 1 case (1.3%), DCIS: 1 case (1.3%)
**Receptor status**	Triple negative: 7 (9.1%), triple positive: 3 (3.9%), hormone receptor positive and HER2 negative: 59 (76.6%), other: 8 (10.4%)
**Focality**	Unifocal: 61 (80.3%), multifocal: 15 (19.7%), missing: 1
**Lymphovascular invasion (LVI)**	Present: 45 (58.4%), absent: 32 (41.6%)

All patients were female and Caucasian. The table summarizes demographic, clinical, pathological, and imaging characteristics, including age, tumor size, nodal burden (pN stage), ultrasound findings, histological subtype, receptor status, focality, and lymphovascular invasion (LVI). Missing data are reported where applicable.

Abbreviations: ER = estrogen, HER2 = human epidermal growth factor receptor 2, PR = progesterone.

### PN1 vs. PN2-3 groups

#### Source of referral

The distribution of referral sources was similar between pN1 and pN2/3 patients. Among those classified as pN1, 25 (75.8%) were symptomatic patients, and 8 (24.2%) were screen-detected. In the pN2/3 group, 35 (79.5%) were symptomatic patients and 9 (20.5%) were screen-detected. There was no statistically significant association between nodal stage and referral type (χ^2^(1) = 0.16, *P = .*692).

#### The number of AUS-detected abnormal nodes

The number of abnormal lymph nodes detected on AUS differed significantly between pN1 and pN2/3 patients. Using the Wilcoxon rank-sum test, the pN2/3 group had a significantly higher number of US-detected abnormal nodes compared to the pN1 group (*z* = -3.91, *P < .*001). The mean number of abnormal nodes was 1.52 (SD 0.89) in the PN1 group and 3.05 (SD 1.85) in the pN2/3 group.

#### Tumor size

Final pathology tumor size was significantly larger in the pN2/3 group compared to the pN1 group. The median difference was statistically significant, as determined by the Wilcoxon rank-sum test (*z* = -3.33, *P < .*001). The mean tumor size in the pN1 group was 36.5 mm (SD 24.3), while the pPN2/3 group had a mean tumor size of 58.8 mm (SD 33.6). These findings suggest an association between larger tumor size and higher axillary nodal burden.

### Diagnostic performance of axillary ultrasound using alternative models

#### Diagnostic performance of axillary ultrasound in differentiating pN1 from pN2/3 disease using a 1-2 abnormal node threshold

The diagnostic performance of AUS in predicting low axillary nodal burden (pN1) was assessed using the presence of 1-2 abnormal lymph nodes on US as the predictive criterion. Only patients with biopsy-confirmed nodal metastases and complete data on both the number of abnormal nodes on US and final pathological nodal status were included (*n = *71).

A confusion matrix summarizing the relationship between US findings (using a 1-2 abnormal node threshold) and pathological nodal burden is presented in Table 2. Among patients with pN1 disease (*n = *31), 27 were correctly identified on US as having 1-2 abnormal nodes (true positives), while 4 were misclassified as having higher nodal involvement (false negatives). Among patients with pN2/3 disease (*n = *40), 22 were correctly classified as not meeting the 1-2 node criterion (true negatives), whereas 18 were incorrectly categorized as pN1 based on their US appearance (false positives).

**Table 2:** Confusion matrix showing the diagnostic performance of axillary ultrasound in predicting pN1 versus pN2/3 disease, using the presence of 1-2 abnormal lymph nodes on ultrasound as the predictive factor.

US result of 1-2 abnormal nodes	pN1 (condition positive)	Not pN1 (condition negative)	Total
**Test positive (1-2 abnormal LN detected)**	TP = 27	FP = 18	45
**Test negative (≥3 abnormal)**	FN* = *4	TN* = *22	26
**Total**	31	40	71

The resulting diagnostic performance of AUS, based on the identification of 1-2 biopsy-confirmed abnormal lymph nodes, demonstrated a sensitivity of 87.1% (95% CI: 70.2-96.4), indicating that 87.1% patients with pN1 disease were correctly identified by this method. The specificity was 55.0% (95% CI: 38.5-70.7), reflecting that 55.0% were correctly identified as not having low-volume disease, pN1. The PPV was 60.0% (95% CI: 44.3-74.3), meaning that when 1-2 abnormal nodes were detected on US, there was a 60% likelihood that the patient truly had limited nodal involvement (pN1). In contrast, the NPV was 84.6% (95% CI: 65.1-95.6), suggesting that patients without this US finding were highly unlikely to have pN1 disease. The overall diagnostic accuracy was 69.0% (95% CI: 56.9-79.5), supporting the potential utility of AUS as a triaging tool in the preoperative assessment of axillary disease burden.

#### Diagnostic performance of axillary ultrasound in differentiating pN1 from pN2/3 disease using a 1 abnormal node threshold

When a stricter threshold of 1 abnormal lymph node on US was used to predict limited nodal disease (pN1), 21 of 31 patients with pN1 disease were correctly identified (true positives), while 10 were misclassified as having higher nodal burden (false negatives). Among patients with pN2/3 disease (*n = *40), 29 were correctly classified as not meeting the single-node criterion (true negatives), whereas 11 were incorrectly categorized as pN1 (false positives). This yielded a sensitivity of 67.7% (95% CI: 48.6-83.3), specificity of 72.5% (95% CI: 56.1-85.4), PPV of 65.6% (95% CI: 46.8-81.4), and NPV of 74.4% (95% CI: 57.9-87.0), with an overall diagnostic accuracy of 70.4% (95% CI: 58.4-80.7). These results indicate that applying a single-node threshold improves specificity and reduces false positives compared with the 1-2 node criterion, but at the expense of lower sensitivity and a higher likelihood of missing true pN1 cases. Table 3 summarizes the diagnostic performance of US using a threshold of 1 abnormal node.

**Table 3.** Confusion matrix showing the diagnostic performance of axillary ultrasound in predicting pN1 versus pN2/3 disease, using the presence of 1 abnormal lymph node on ultrasound as the predictive factor.

US result	pN1 (condition positive)	Not pN1 (condition negative)	Total
**US positive (1 abnormal LN)**	TP = 21	FP = 11	32
**US negative (>1 abnormal LN)**	FN* = *10	TN* = *29	39
**Total**	31	40	71

#### Diagnostic performance of ultrasound using the (1-2 abnormal nodes as the threshold and tumor size of ≤5 cm on ultrasound)

The diagnostic performance of US in predicting axillary tumor burden was evaluated using a logistic regression model incorporating the number of abnormal nodes detected on US (>2 vs. 1-2), tumor size (>5 cm vs. ≤5 cm), and their interaction.

The model was statistically significant (LR χ^2^(3) = 14.07, *P = .*0028) and demonstrated an overall pseudo *R*^2^ of 0.133, indicating that approximately 13% of the variation in nodal burden was explained by the predictors (Table S1). Discriminative ability was acceptable, with an area under the ROC curve (AUC) of 0.73, while calibration was excellent (Hosmer-Lemeshow *P* = 1.00).

Table 4 summarizes the effects of individual predictors. Patients with more than 2 abnormal nodes on US had 6-fold higher odds of harboring high-burden nodal disease (pN2/3) compared with those with 1 to 2 abnormal nodes (OR = 6.0, 95% CI: 1.73-20.82, *P = .*005). Tumor size greater than 5 cm showed a non-significant trend toward higher nodal burden (OR = 2.8, 95% CI: 0.72-10.87, *P = .*14), and the interaction between node count and tumor size was not significant (*P = .*65), indicating that the effect of US findings on nodal stage was independent of tumor size.

**Table 4:** Odds ratios (ORs) and 95% confidence intervals (CIs) from multivariable logistic regression examining predictors of high-burden nodal disease (pN2/3).

Predictor	Odds ratio (OR)	95% CI	*P* value	Interpretation
**>2 abnormal nodes on US**	6.0	1.73-20.82	.005	Strong, independent predictor. Patients with >2 abnormal nodes on ultrasound have 6× higher odds of pN2/3 disease than those with 1-2 nodes.
**Tumor size > 5 cm**	2.8	0.72-10.87	.137	Trend toward higher nodal burden with larger tumors, but not statistically significant.
**Interaction (>2 nodes × >5 cm)**	0.60	0.06-5.78	.655	No evidence of synergistic interaction—effect of US node count does not depend on tumor size.

“>2 abnormal nodes” refers to ultrasound detection of more than 2 suspicious axillary nodes; “>5 cm” denotes pre-operative tumor size greater than 5 cm. The interaction term assesses whether the effect of ultrasound findings varies by tumor size. A *P* value <.05 was considered statistically significant.

These results highlight US-detected node count as the dominant predictor of axillary disease extent, while tumor size adds limited incremental prognostic value.

Model classification statistics are presented in Table 5. At a .5 probability threshold, the model correctly classified 70.1% of patients, with a sensitivity of 74.4% (95% CI: 67.2-92.7), specificity of 64.7% (95% CI: 54.0-80.5), PPV of 72.7% (95% CI: 52.5-84.9), and NPV of 66.7% (95% CI: 56.9-79.5). These findings indicate good overall diagnostic performance in identifying patients with extensive nodal disease, with balanced accuracy and acceptable discriminative ability.

**Table 5:** Diagnostic performance of the combined logistic regression model in predicting pN2/3 disease.

Metric	Value	Interpretation
**Sensitivity (true positive rate)**	74.4%	Correctly identified 74% of pN2/3 cases.
**Specificity (true negative rate)**	64.7%	Correctly identified 65% of pN1 cases.
**Positive predictive value (PPV)**	72.7%	When model predicts pN2/3, it is correct ∼73% of the time.
**Negative predictive value (NPV)**	66.7%	When model predicts pN1, it is correct ∼67% of the time.
**Overall accuracy**	70.1%	Correct classification in 7 out of 10 patients.

## Discussion

This study demonstrates the valuable role of preoperative AUS, coupled with core needle biopsy, in stratifying nodal burden in patients with early invasive breast cancer.

Our findings indicate that the number of abnormal nodes detected via US is significantly associated with the eventual pathological nodal burden. Patients with lower abnormal nodal counts on US were found to have low-volume disease, as reflected in the mean nodal burden of 1.52 nodes in pN1 cases, compared with a higher mean of 3.05 nodes in pN2-3 disease. This finding is consistent with previous studies, which similarly reported a correlation between the US-detected nodal burden^17^^,^  ^19^^,^^20^ tumor load effectively. Furthermore, using univariate analysis, tumor size emerged as another significant predictor, with larger tumors correlating strongly with higher nodal disease stages (pN2-3). The relatively large mean tumor size of 49 mm in our cohort could reflect the small sample size and or the local criteria adopted for classifying a lymph node as abnormal on US. But the association between tumor size and nodal involvement has been consistently reflected in the literature.^21–26^

The diagnostic accuracy of US in identifying patients with pN1 disease in our study is similar to previous studies.^15^^,^  ^17^ Our study demonstrated that AUS demonstrated good performance in identifying patients with limited nodal disease (pN1), but its ability to discriminate higher axillary burden (pN2/3) was more limited. This finding underscores the need for careful interpretation of AUS nodal counts within the broader clinical and pathological context.

The predictive value of nodal counts on AUS could be interpreted in conjunction with primary tumor characteristics, particularly tumor size. A large population-level study shows a rising probability and burden of nodal metastasis with increasing tumor size.^24^ Incorporating tumor size into preoperative assessment can refine risk stratification and reduce false-positive interpretations, improving selection for less extensive axillary surgery. Moreover, imaging studies that evaluate both tumor size and the number of suspicious nodes on axillary AUS report that these are independent predictors of high axillary burden (≥3 involved nodes), and that combining them improves discrimination.^27^ For example, AUS showing ≥2 highly suspicious nodes plus tumor >10 mm yields a high PPV for pN2+ disease.^27^

In our study, when considering which US-based approach would support risk stratification, both the single-node model and the logistic regression model demonstrated favorable and comparable performance. The model using a threshold of 1 abnormal lymph node on US achieved balanced diagnostic accuracy, with a sensitivity of 67.7% and specificity of 72.5%, resulting in an overall accuracy of 70.4%. This approach substantially reduced false positives compared with the 1-2 node criterion, thereby lowering the likelihood of incorrectly classifying patients with extensive nodal involvement (pN2/3) as having low-burden disease. Similarly, the logistic regression model that incorporated US node count and tumor size achieved an almost identical classification accuracy of 70.1%, with a sensitivity of 74.4% and specificity of 64.7%, and demonstrated acceptable discrimination (AUC = 0.73).

Taken together, these results indicate that either the single-node US model or the logistic regression model can be used effectively to identify patients with low-burden axillary disease. Both approaches provide comparable accuracy and predictive performance, while maintaining an acceptable balance between sensitivity and specificity. The single-node model may be more practical for routine clinical application due to its simplicity, whereas the logistic regression model offers greater flexibility for centers using predictive modeling to refine TAD eligibility criteria.

Contemporary axillary management for low-burden node-positive disease remains heterogeneous. Despite evidence supporting de-escalation, most UK breast units continue to perform ALND for biopsy-proven nodal metastases identified preoperatively. A recent national survey reported that 78.5% of centers still perform full ALND in patients with ≤2 suspicious nodes on US, while only 15.7% have adopted TAD routinely or selectively.^12^ This variation reflects persistent uncertainty regarding the reliability of preoperative axillary staging tools. The audit to quantify the volume of disease in the axilla on US (the AVOID study) is addressing 1 important facet of this uncertainty: the threshold for what constitutes an “abnormal” node on US.^28^ This audit’s key aims include describing current UK practice in terms of the cortical thickness threshold used to prompt biopsy and assessing the association between cortical thickness thresholds and the actual volume of malignant axillary disease at dissection.^28^ By standardizing and collecting national-level data on cortical thickness and node counts, this audit may help clarify optimal US criteria for axillary staging and thereby reduce variability in clinical practice.^28^ The findings could significantly inform surgical planning, particularly by identifying patients with limited nodal involvement who may be considered for less invasive axillary management strategies.^28^

Our study shows that using a 3-mm threshold for cortical thickness or destruction of the nodal architecture on imaging as criteria for identifying a suspicious lymph node, allows successful risk stratification. In our unit, the threshold of a cortical thickness exceeding 3 mm was selected in accordance with the Royal College of Radiologists (RCR) Breast Imaging Guidelines, 4th edition, which note that there is no nationally agreed cutoff and that reported thresholds vary between 2 and 4 mm across centers.^29^ This threshold is routinely applied and locally audited to ensure an appropriate balance between detection of low-volume nodal disease and avoidance of unnecessary axillary clearances. Additionally, only patients with biopsy-confirmed abnormal lymph nodes were included in the study. Lloyd et al^30^ support this stratification approach, illustrating that many patients with biopsy-proven positive nodes on US present with limited axillary disease, thereby questioning the necessity of routine ALND. Similarly, another study reported that more than half (53%) of those with screen-detected breast cancer proceeding directly to ALND after a positive needle core biopsy actually had low nodal burden (≤2 positive nodes) at final pathology.^31^ Another similar observation in a different study reinforces this perspective, arguing against obligatory axillary clearance in cases demonstrating minimal nodal involvement preoperatively.^32^

Finally, our study facilitates the adoption of targeted axillary dissection (TAD), an approach increasingly validated by prospective studies. Combining sentinel lymph node biopsy with removal of the clipped node after neoadjuvant chemotherapy has been shown to significantly reduce false-negative rates compared with sentinel biopsy alone, establishing TAD as a feasible staging technique.^33^ Ongoing multicenter randomized trials are now evaluating whether TAD can safely replace axillary lymph node dissection (ALND) in selected patients with a good response to neoadjuvant therapy. Among these, the Axillary Treatment in women with breast cancer with lymph Node positive disease after Neo-adjuvant Chemotherapy (ATNEC) trial is particularly noteworthy.^34^ The ATNEC trial randomizes patients with biopsy-proven node-positive breast cancer who achieve complete pathological nodal response after neoadjuvant chemotherapy to either no treatment or treatment in the form of ALND or ART.^34^

A key consideration when interpreting this study’s finding is the distinction between predictive stratification and treatment decision-making. This study specifically demonstrates the ability of AUS to predict and stratify axillary nodal burden in patients with biopsy-proven nodal disease. However, it does not address the oncological safety or outcome-based replacement of ALND with TAD, which requires validation through prospective clinical trials. Accordingly, AUS should be considered a tool for preoperative risk assessment to support multidisciplinary decision-making, rather than as a standalone determinant of treatment de-escalation.

Despite the strengths of this study, some limitations must be considered when interpreting our findings. This is a retrospective, single-centre study with a modest sample size, which may affect statistical robustness. This introduces uncertainty into the estimated diagnostic performance metrics. Although sensitivity and NPV were relatively high, the associated confidence intervals are wide, particularly for specificity and predictive values, reflecting limited precision. These findings should therefore be interpreted with caution, and further validation in larger prospective cohorts is required. Subgroup analysis by tumor subtype was not feasible due to the limited sample size within each histological category. The operator-dependent nature of AUS also introduces variability in node assessment. Furthermore, multiple US-based thresholds and predictive models were explored, which introduces a risk of type I error inflation. These analyses should therefore be considered exploratory, and the findings, particularly marginal or non-significant associations, should be interpreted with caution. In addition, the study population was restricted to biopsy-proven node-positive patients who underwent axillary lymph node dissection, representing a selected higher-risk subgroup. This may introduce spectrum bias and influence the observed diagnostic performance, limiting generalizability to broader breast cancer populations, particularly those with clinically node-negative disease or those managed with less invasive axillary approaches. Moreover, all patients in this cohort were Caucasian women, which may further limit the generalizability of these findings to more ethnically diverse populations.

In conclusion, this recent study based on real-world evidence affirms the utilization of preoperative AUS combined with biopsy confirmation in stratifying axillary nodal disease burden. Importantly, our data reveals that while AUS performs well in identifying pN1 disease, it remains equivocal in detecting pN2/3 disease, with a significant false-negative rate. Therefore, we propose that AUS findings should be integrated with other relevant parameters rather than be used in isolation. Further prospective research is needed to refine imaging criteria and explore adjunct modalities to enhance the detection of high-burden disease. By providing additional evidence in a clearly defined cohort, this study supports the role of AUS in preoperative risk stratification and may help inform patient selection for less invasive axillary management strategies alongside other factors.

## Supplementary Material

tqag145_Supplementary_Data

## Data Availability

The data are available from the corresponding author upon reasonable request.
