# Supplementary material for: Less is more: the role of preoperative ultrasound as a risk stratifying tool in identifying low volume axillary disease in breast cancer
Source: Br J Radiol. 2026 Jun 9;99(1186):2178–86. doi: 10.1093/bjr/tqag145 (PMC13623215; doi:10.1093/bjr/tqag145)
Supplement: Supplementary file 1 [file tqag145_supplementary_data.zip › Supplemantary Table 1.docx]

| **Statistic** | **Result** | **Interpretation** |
| --- | --- | --- |
| **N (observations)** | 77 | Adequate sample for a 2–3 variable logistic model |
| **LR χ² (3)** | 14.07 | Model statistically significant |
| **p-value** | 0.0028 | Predictors jointly improve fit vs. null model |
| **Pseudo R²** | 0.133 | Explains ~13% of variability in nodal burden |
| **AUC (ROC)** | 0.7285 | Acceptable discrimination |
| **Hosmer–Lemeshow p** | 1.000 | Excellent calibration (no lack of fit) |

***Supplementary Table 1***. *Logistic regression model assessing the predictive value of ultrasound node count (>2 vs 1–2 abnormal nodes), tumour size (>5 cm), and their interaction in identifying high-burden axillary disease (pN2/3). Model statistics include likelihood ratio chi-square, pseudo-R², overall significance (p < 0.05 indicates model improvement over null), area under the ROC curve (AUC) for discrimination, and Hosmer–Lemeshow goodness-of-fit (p > 0.05 indicates adequate calibration).*
